# Epithelial–Mesenchymal Transition Markers in Clear Cell Renal Cell Carcinoma: Expression Patterns and Prognostic Significance

**DOI:** 10.3390/jpm16060279

**Published:** 2026-05-24

**Authors:** Lara Smoljo, Tonka Mateljak, Anita Racetin, Petar Todorović, Jelena Komić, Luka Komić, Petar Đolonga, Danijel Antonio Grubišić, Sandra Kostić, Katarina Vukojević, Nela Kelam

**Affiliations:** 1 Department of Anatomy, Histology and Embryology, Laboratory for Early Human Development, University of Split School of Medicine, Šoltanska 2A, 21000 Split, Croatia; lara.smoljo@mefst.hr (L.S.); tonka.mateljak@mefst.hr (T.M.); anita.racetin@mefst.hr (A.R.); petar.todorovic@mefst.hr (P.T.); katarina.vukojevic@mefst.hr (K.V.); nela.kelam@mefst.hr (N.K.); 2 Center for Translational Research in Biomedicine, University of Split School of Medicine, Šoltanska 2A, 21000 Split, Croatia; 3 Department of Family Medicine, Split-Dalmatia County Health Center, 21000 Split, Croatia; jelena.kelam@mefst.hr (J.K.); luka.komic@mefst.hr (L.K.); 4 Department of Pathology, Forensic Medicine and Cytology, University Hospital of Split, 21000 Split, Croatia; petar.dolonga@mefst.hr (P.Đ.); danijel-antonio.grub@kbsplit.hr (D.A.G.); 5 Mediterranean Institute for Life Sciences (MedILS), University of Split, Meštrovićevo Šetalište 45, 21000 Split, Croatia

**Keywords:** clear cell renal cell carcinoma, epithelial–mesenchymal transition, EMT, *PCDH9*, *CTNNB1*, *β-catenin*, *vimentin*, *VIM*, *SNAI1*

## Abstract

**Background/Objectives:** Clear cell renal cell carcinoma (ccRCC) is the most prevalent subtype of renal cancer, characterized by frequent metastasis and poor prognosis. Epithelial–mesenchymal transition (EMT) plays a pivotal role in tumor progression. Protocadherin 9 (PCDH9) has emerged as a potential tumor suppressor, but its relationship with EMT markers in ccRCC remains unclear. This study aimed to investigate the expression patterns and prognostic significance of PCDH9, β-catenin (CTNNB1), Snail (SNAI1), and Vimentin (VIM) in ccRCC. **Methods:** Immunofluorescence analysis was performed on formalin-fixed paraffin-embedded tissue sections from 48 ccRCC patients (31 low-grade, 17 high-grade) and adjacent normal renal cortex. Findings were validated using The Cancer Genome Atlas (TCGA-KIRC) dataset via GEPIA2/GEPIA3 platforms, including differential expression, correlation, and survival analyses. **Results:** *PCDH9* mRNA was significantly downregulated in ccRCC tumors (TCGA-KIRC), while *VIM* was upregulated at the transcriptomic level. Tissue-level immunofluorescence quantification revealed discordant patterns, highlighting the influence of cellular heterogeneity on bulk protein assessment. The strong positive correlation between *PCDH9* and *CDH1* observed in normal kidney was completely lost in tumor tissue. Unexpectedly, *PCDH9* showed positive correlations with EMT transcription factors (*ZEB1*, *SNAI1*) in tumors. In univariate survival analysis, high *PCDH9* and *CTNNB1* expression were associated with improved overall survival. Multivariate Cox regression revealed endpoint-specific prognostic signatures: *VIM* independently predicted disease progression, while *SNAI1* predicted overall mortality. *CTNNB1* was consistently protective across both endpoints. **Conclusions:** Our findings support a tumor-suppressive role for *PCDH9* in ccRCC and reveal disruption of epithelial adhesion molecule co-regulation during tumorigenesis. The identification of endpoint-specific prognostic signatures has implications for patient stratification and suggests that ccRCC exhibits a partial EMT phenotype rather than classical EMT.

## 1. Introduction

Clear cell renal cell carcinoma (ccRCC) represents the most prevalent subtype of renal cell carcinoma, accounting for approximately 70–80% of all cases [1,2,3]. Despite significant advances in therapeutic approaches, including targeted therapies and immunotherapy, ccRCC remains clinically challenging due to its frequent diagnosis at advanced stages and limited response to conventional chemotherapy [4,5]. The five-year survival rate for metastatic ccRCC remains poor, underscoring the urgent need for a better understanding of the molecular mechanisms driving tumor progression and metastasis [2].

Epithelial-to-mesenchymal transition (EMT) has emerged as a pivotal process in the progression and metastatic dissemination of solid tumors, including ccRCC [6,7]. During EMT, epithelial cells lose their intercellular junctions and apical-basal polarity while acquiring mesenchymal characteristics that enable invasion and metastasis [7,8]. This phenotypic plasticity is orchestrated by a network of transcription factors, adhesion molecules, and signaling pathways that coordinate the downregulation of epithelial markers and upregulation of mesenchymal markers [9,10].

Protocadherin 9 (PCDH9) is a member of the protocadherin family and is increasingly recognized as a potential tumor suppressor in various malignancies [11,12]. Previous studies have demonstrated that *PCDH9* expression is reduced in numerous carcinomas and that its loss correlates with poorer prognosis [11,12]. *PCDH9* regulates cell adhesion properties and can influence the β-catenin signaling pathway, which serves as a central regulator of EMT [13]. When β-catenin is not bound to E-cadherin at the cell membrane, it can translocate to the nucleus, where it acts as a transcriptional co-activator and promotes the expression of EMT-related genes, including the transcription factor *Snail* [14,15,16]. *Snail* subsequently suppresses the expression of epithelial markers and induces the expression of mesenchymal markers such as vimentin [17,18,19].

The interconnection between *PCDH9*, *β-catenin*, and downstream EMT effectors in ccRCC has not been systematically investigated. Understanding these relationships is particularly relevant given that ccRCC exhibits constitutive activation of hypoxia-inducible factors due to frequent *VHL* mutations, which can further modulate EMT-related pathways [20,21,22,23]. Moreover, the prognostic significance of these markers and their potential utility in risk stratification of ccRCC patients warrant further exploration.

The aim of this study was to examine the expression and localization of PCDH9, β-catenin (CTNNB1), Snail (SNAI1), and Vimentin (VIM) in ccRCC samples of different histological grades and to investigate their interrelationships in the context of EMT. We employed immunofluorescence staining on tissue samples from patients with low-grade and high-grade ccRCC, complemented by bioinformatic analysis of The Cancer Genome Atlas (TCGA) kidney renal clear cell carcinoma (KIRC) dataset to validate our findings at the transcriptomic level and assess their prognostic significance. We hypothesized that reduced PCDH9 expression is associated with nuclear translocation of β-catenin, elevated expression of Snail and Vimentin, and higher tumor grade, collectively reflecting activation of the EMT program in aggressive ccRCC.

## 2. Materials and Methods

### 2.1. Sample Collection and Processing

The study cohort comprised 48 patients with histopathologically confirmed clear cell renal cell carcinoma (ccRCC). Formalin-fixed, paraffin-embedded (FFPE) tissue blocks from nephrectomy specimens were retrieved from the archival collection of the Department of Pathology, Forensic Medicine and Cytology, University Hospital of Split, covering the period from 2023 to 2024. For each patient, both tumor tissue and adjacent histologically normal renal cortex were collected, with the latter serving as an internal control. Inclusion criteria required sufficient FFPE material for immunofluorescence analysis and complete accompanying clinical documentation. Cases with incomplete pathological data or inadequate tissue material were excluded from the study. The study was conducted in accordance with the Declaration of Helsinki. It was approved by the Ethics Committee of the University Hospital Center Split (class: 500-03/23-01/187, protocol code no. 2181-147-01-06/LJ.Z.-23-02, date of approval: 20 September 2023) and the Ethics Committee of the University of Split, School of Medicine (class: 003-08/23-03/0015, protocol code no.: 2181-198-03-04-23-0073, date of approval: 27 September 2023). Tumor grading was assessed according to the 2016 World Health Organization/International Society of Urological Pathology (WHO/ISUP) consensus system [24]. The cohort included 7 patients classified as grade 1 (G1), 24 as grade 2 (G2), 14 as grade 3 (G3), and 3 as grade 4 (G4), reflecting the natural distribution of tumor grades encountered in clinical practice. For comparative analyses, tumors were additionally grouped into low-grade (G1 + G2, *n* = 31) and high-grade (G3 + G4, *n* = 17) categories. The mean patient age was 68.7 ± 11.3 years, with a male predominance (33/48, 68.8%). All tumors were of clear cell histological subtype and predominantly unifocal (45/48, 93.8%).

For immunofluorescence analysis, adjacent histologically normal renal cortex served as control tissue. Because paired tumor and control tissue were obtained from the same nephrectomy specimen, the availability of a well-preserved cortical rim varied across cases depending on the extent of tumor involvement and the orientation of the tissue block. Consequently, not every tumor sample had a corresponding control section on the same slide. Control tissue was identified by an experienced pathologist as morphologically normal renal cortex located at the periphery of the specimen, and was included in the analysis only when a clearly identifiable cortical rim was present at the edge of the tissue section. This approach resulted in variable control group sizes across staining panels (*n* = 13–17), as the number of specimens with adequate cortical tissue differed between the two antibody combinations (PCDH9/β-catenin and SNAI1/VIM).

Archival nephrectomy specimens had been routinely processed in the pathology laboratory using standard histological procedures, including fixation in 4% paraformaldehyde (PFA) in 0.1 M phosphate-buffered saline (PBS), dehydration through a graded ethanol series, paraffin embedding, and serial sectioning at 5 μm thickness. Sections were mounted onto glass slides for subsequent hematoxylin–eosin (H&E) staining and immunofluorescence analysis.

### 2.2. Immunofluorescence Staining

Following deparaffinization in xylene and stepwise rehydration in ethanol, sections underwent heat-induced epitope retrieval in 0.01 M citrate buffer (pH 6.0) at 95 °C for 30 min in a water steamer. After equilibration to room temperature, slides were rinsed in 0.1 M PBS and pre-treated for 20 min with a protein blocking reagent (ab64226, Abcam, Cambridge, UK) to suppress nonspecific binding.

Primary antibodies were applied to tissue sections and incubated overnight in a humidity-controlled chamber (Table 1). Following overnight incubation, slides were washed with PBS and incubated with corresponding fluorophore-conjugated secondary antibodies for one hour at room temperature (Table 1). Nuclear counterstaining was performed with 4′,6-diamidino-2-phenylindole (DAPI) following a final PBS wash. Sections were air-dried and mounted using Immuno-Mount medium (Thermo Shandon, Pittsburgh, PA, USA) to preserve fluorescent signals for microscopic evaluation.

Antibody specificity was confirmed through preadsorption controls, in which each primary antibody was preincubated with the corresponding peptide antigen prior to application. Under these conditions, no immunoreactivity was detected. Additionally, omission of primary antibodies during the staining protocol yielded no detectable signal, confirming the absence of nonspecific secondary antibody binding.

### 2.3. Imaging and Quantitative Image Analysis

Histological evaluation of H&E-stained sections was performed using a light microscope (BX51, Olympus, Tokyo, Japan). Immunofluorescence imaging of renal tissue was performed using a BX51 Olympus fluorescence microscope equipped with a Nikon DS-Ri2 digital camera (Nikon Corporation, Tokyo, Japan) and NIS-Elements F software (version 3.0). For each tissue section, ten non-overlapping representative fields were captured at ×40 magnification with constant gain and exposure settings across all samples.

Quantitative analysis of immunofluorescent signal was performed using ImageJ software (version 1.54g; NIH, Bethesda, MD, USA). The proportion of each microscopic field occupied by positive signal (area percentage) was determined after median-filter background subtraction and intensity-based thresholding, in line with previously validated workflows from our group [25,26,27]. In practical terms, the area percentage corresponds to the fraction of pixels above a defined intensity threshold relative to the total pixels of the analyzed region. Measurements were obtained on a per-field basis without segmenting individual cell populations, and per-sample values were expressed as the mean across the captured fields.

Co-localization between paired antibodies (PCDH9/β-catenin and SNAI1/VIM) was quantified at two complementary levels. First, pixel-level co-localization was performed on five representative double-stained fields per group (CTRL, low-grade, and high-grade ccRCC; *n* = 15 fields per antibody pair) using the JACoP plugin [28] in ImageJ v1.54g. For each field, the green and red channels were split, background was subtracted (rolling-ball, radius 50 px), and Costes’ automatic thresholding was applied independently to each channel. Pearson’s correlation coefficient (PCC) and Manders’ overlap coefficients M1 (fraction of green signal co-localizing with red) and M2 (fraction of red signal co-localizing with green) were computed. Costes’ randomisation (200 iterations) was applied to confirm that the observed co-localization significantly exceeded that expected from random pixel distributions. Second, tissue-level (per-case) co-expression was assessed by computing Spearman’s rank correlation coefficient (ρ) between the per-case area-percentages of paired markers within each group.

To minimize interobserver variability, three experienced histologists independently evaluated all captured microphotographs and set background thresholds using negative control images. Interrater agreement was assessed by intraclass correlation analysis, yielding a coefficient greater than 0.80, indicating excellent concordance [29].

### 2.4. Statistical Analysis

Normality of data distribution was assessed using the Shapiro–Wilk test for each marker and group. For SNAI1, VIM, and CTNNB1, one or more groups showed significant departures from normality; therefore, nonparametric tests were applied. For PCDH9, all groups satisfied the normality assumption, and parametric tests were used. Immunoexpression data are presented graphically as box-and-whisker plots (median, interquartile range, and minimum/maximum values), with individual data points superimposed for all analyzed samples.

For SNAI1, VIM, and CTNNB1, immunoexpression was compared at three levels. Differences between histologically normal renal cortex (CTRL) and pooled ccRCC tissue (all grades combined) were assessed using the Mann–Whitney U test (two-tailed). To evaluate differences by grade category, immunoexpression was compared among CTRL, low-grade ccRCC, and high-grade ccRCC using the Kruskal–Wallis test, followed by Dunn’s post hoc test for pairwise multiple comparisons when a significant overall difference was detected. Similarly, comparisons across all individual tumor grades were performed using the Kruskal–Wallis test with Dunn’s post hoc correction.

For PCDH9, differences between CTRL and pooled ccRCC tissue were assessed using Welch’s *t*-test (two-tailed, not assuming equal variances). Comparisons among CTRL, low-grade, and high-grade groups, and across individual tumor grades, were performed using ordinary one-way ANOVA followed by Tukey’s post hoc test for pairwise multiple comparisons.

For the analysis of clinicopathological characteristics, differences in continuous variables (age, tumor size) across tumor grades were assessed using the Kruskal–Wallis test, while comparisons between two groups (low-grade vs. high-grade) were performed using the Mann–Whitney U test (two-tailed). Categorical variables (sex, laterality, pT stage, and focality) were compared across tumor grades using the chi-square test or Fisher’s exact test, as appropriate. Correlations between continuous variables (tumor size, age) and tumor grade were evaluated using Spearman’s rank correlation coefficient (ρ). The association between immunoexpression levels and patient age was assessed using Spearman’s correlation, and sex-based differences in protein expression were evaluated using the Mann–Whitney U test.

All graphical representations were generated in GraphPad Prism 10.6.1, and illustrative figure panels were assembled using Adobe Photoshop v21.0.2 (Adobe Systems, San Jose, CA, USA).

### 2.5. Bioinformatic Analysis of Public Datasets

#### 2.5.1. Data Source

Gene expression data for *PCDH9*, *SNAI1*, *CTNNB1*, and *VIM* in clear cell renal cell carcinoma were obtained from The Cancer Genome Atlas Kidney Renal Clear Cell Carcinoma (TCGA-KIRC) dataset. The TCGA-KIRC cohort includes 531 tumor samples and 72 matched peritumor (adjacent normal) samples. For comparison with healthy tissue, normal kidney cortex samples from the Genotype-Tissue Expression (GTEx) project (*n* = 28) were also included where applicable.

#### 2.5.2. Differential Expression Analysis

Differential mRNA expression between tumor (*n* = 523) and normal *(n* = 100) kidney tissue was assessed using the Gene Expression Profiling Interactive Analysis 2 (GEPIA2) platform (GEPIA2; http://gepia2.cancer-pku.cn/, 27 February 2026) [30]. The “Multiple Genes Comparison” module with box plot visualization was used. Gene expression levels of *PCDH9*, *SNAI1*, *CTNNB1*, and *VIM* were compared between tumor and normal tissues using one-way ANOVA. Expression values were log_2_-transformed (TPM + 1) prior to analysis. Differential expression was considered statistically significant when |log_2_FC| ≥ 1 and *p*-value < 0.01.

#### 2.5.3. Correlation Analysis

Pairwise gene expression correlations were performed using the “Correlation Analysis” module of Gene Expression Profiling Interactive Analysis 3 (GEPIA3) online platform (http://gepia3.bioinfoliu.com/, 27 February 2026). Correlations were calculated between *PCDH9* and genes representing epithelial–mesenchymal transition (EMT) markers (*CDH1*, *SNAI1*, *SNAI2*, *VIM*, *ZEB1*, *ZEB2*, *TWIST1*, *CDH2*); Wnt/β-catenin signaling pathway components (*CTNNB1*, *AXIN2*, *MYC*, *CCND1*, *LEF1*), and ccRCC-specific markers (*VHL*, *HIF1A*, *CA9*). As internal controls, correlations between established EMT marker pairs (*CDH1*-*SNAI1*, *CDH1*-*VI*M) were also assessed. Spearman’s rank correlation coefficient (ρ) was used to evaluate the strength and direction of associations. Gene expression values were log_2_-transformed (TPM + 1) prior to analysis. Analyses were performed separately for three tissue types: KIRC tumor (*n* = 531), KIRC peritumor (*n* = 72), and GTEx normal kidney (*n* = 28). Statistical significance was set at *p* < 0.05.

#### 2.5.4. Univariate Survival Analysis

Univariate survival analysis was performed using the “Survival Analysis” module of GEPIA2. The TCGA-KIRC cohort was stratified into high- and low-expression groups based on the median expression value for each gene (cutoff: 50th percentile). Two survival endpoints were analyzed: overall survival (OS), defined as the time from diagnosis to death from any cause, and disease-free survival (DFS), defined as the time from diagnosis to disease recurrence. Survival curves were generated using the Kaplan–Meier method, and differences between groups were assessed using the log-rank test. Hazard ratios (HR) with 95% confidence intervals were calculated to quantify the relative risk associated with high versus low gene expression. Statistical significance was set at *p* < 0.05. The analysis included approximately 508–516 patients for OS (*n* (high) = 256–258, *n* (low) = 252–258) and 516 patients for DFS (*n* (high) = 258, *n* (low) = 257–258), depending on data availability for each gene.

#### 2.5.5. Multivariate Survival Analysis

Multivariate Cox proportional hazards regression analysis was performed using the Gene Expression Profiling Interactive Analysis 3 (GEPIA3) platform (http://gepia3.cancer-pku.cn/, 27 February 2026) [31], to evaluate the independent prognostic value of each gene while controlling for the effects of the other genes. The “TimeResponse” module was used with the TCGA-KIRC dataset. Two survival endpoints were analyzed: overall survival (OS) and progression-free interval (PFI), the latter defined as the time from diagnosis to disease progression or recurrence. All four genes (*PCDH9*, *SNAI1*, *CTNNB1*, *VIM*) were included simultaneously in each Cox regression model. Hazard ratios (HRs) with 95% confidence intervals (CIs) were calculated; HR > 1 indicates increased risk (poor prognosis), and HR < 1 indicates decreased risk (favorable prognosis). Statistical significance was set at *p* < 0.05.

## 3. Results

### 3.1. Clinical and Pathological Characteristics of the Study Cohort

The study included 48 patients with histopathologically confirmed ccRCC, comprising 33 males (68.8%) and 15 females (31.2%), with a mean age of 68.7 ± 11.3 years (median 72, range 39–87 years). All tumors were of clear cell histological subtype and predominantly unifocal (45/48, 93.8%). According to the WHO/ISUP grading system, the cohort included 7 G1, 24 G2, 14 G3, and 3 G4 tumors. For comparative analyses, tumors were additionally classified as low-grade (G1 + G2, *n* = 31) or high-grade (G3 + G4, *n* = 17).

No statistically significant differences in patient age (Kruskal–Wallis, H = 2.14, *p* = 0.545), sex distribution (chi-square, χ^2^ = 0.125, *p* = 0.989), or tumor laterality (chi-square, χ^2^ = 2.35, *p* = 0.504) were observed across tumor grades (Table 2), confirming that the groups were demographically comparable. Similarly, no significant differences in age (Mann–Whitney U test, *p* = 0.164) or sex (Fisher’s exact test, *p* = 1.000) were found between the low-grade and high-grade groups (Table 3).

In contrast, maximum tumor diameter differed significantly across tumor grades (Kruskal–Wallis, H = 11.52, *p* = 0.009), with a significant positive correlation between tumor grade and tumor size (Spearman ρ = 0.473, *p* < 0.001). Mean tumor size increased progressively from 4.0 ± 2.3 cm in G1 to 4.7 ± 1.9 cm in G2, 7.8 ± 4.6 cm in G3, and 11.0 ± 2.0 cm in G4. High-grade tumors were significantly larger than low-grade tumors (8.3 ± 4.4 vs. 4.5 ± 2.0 cm; Mann–Whitney U test, *p* = 0.004; Table 2). Furthermore, all three G4 tumors were classified as pT3a, whereas the majority of G1 tumors (6/7, 85.7%) were confined to pT1. Complete clinicopathological characteristics are summarized in Table 2, and the comparison between low-grade and high-grade groups is presented in Table 3.

### 3.2. Histological Evaluation of Normal Kidney Tissue and Clear Cell Renal Cell Carcinoma Across Tumor Grades

Clear cell renal cell carcinoma originates from epithelial cells of the proximal tubules. Different grades (G) of ccRCC were studied. Histological examination of H&E-stained kidney sections revealed characteristic features distinguishing control renal tissue from various tumor grades of ccRCC.

**Figure 1:**
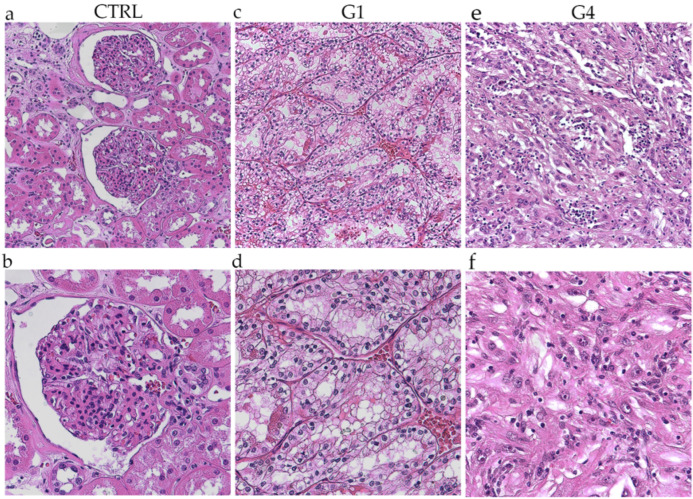
Histological evaluation of control renal cortex and clear cell renal cell carcinoma (ccRCC) by hematoxylin–eosin (H&E) staining. (**a**,**b**) Control kidney tissue showing preserved renal architecture with well-defined glomeruli and tubules; tubular epithelial cells display uniform nuclei with finely dispersed chromatin and inconspicuous nucleoli. (**c**,**d**) Grade 1 (G1) ccRCC with clear cytoplasm, small round nuclei, and minimal pleomorphism; nucleoli are indistinct and visible only at higher magnification. (**e**,**f**) Grade 4 (G4) ccRCC showing disorganized architecture, enlarged irregular nuclei with prominent nucleoli, moderate nuclear pleomorphism, and increased stromal cellularity with inflammatory infiltrates. Upper panels: ×200 magnification; lower panels: ×400 magnification.

In the control group, normal renal architecture was preserved, with well-defined glomeruli and renal tubules (Figure 1a,b). The tubular epithelial cells exhibited uniform nuclei with finely dispersed chromatin and inconspicuous nucleoli (Figure 1b). No cellular atypia or pathological alterations were observed (Figure 1a,b).

In Grade 1 ccRCC samples, the neoplastic cells demonstrated clear cytoplasm, consistent with glycogen and lipid accumulation (Figure 1c,d). The nuclei were small, round, and uniform, with indistinct nucleoli visible only at high magnification (400×, Figure 1d). The overall architecture remained partially preserved, and nuclear pleomorphism was minimal (Figure 1d). In contrast, G4 ccRCC tissues exhibited a more disorganized architecture (Figure 1e,f). Tumor cells retained clear cytoplasm, but nuclei were larger, with irregular contours and prominent nucleoli visible at high magnification (400×, Figure 1f). Moderate nuclear pleomorphism was evident, and stromal regions showed increased cellularity with occasional inflammatory infiltrates (Figure 1e).

**Figure 2:**
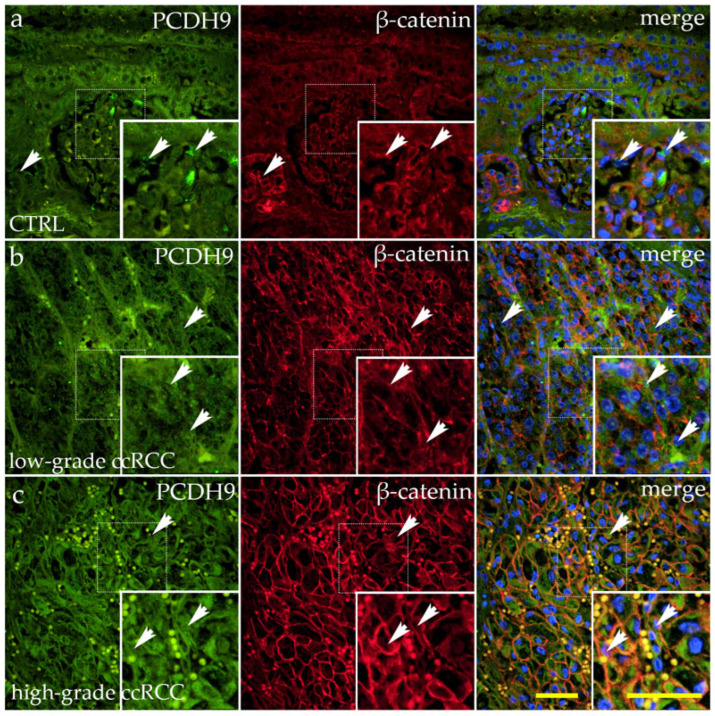
Representative double immunofluorescence micrographs showing the expression and co-localization of PCDH9 (green) and β-catenin (red) in control renal cortex and clear cell renal cell carcinoma (ccRCC). Nuclei were counterstained with DAPI (blue). (**a**) Control kidney tissue (CTRL): PCDH9 immunoreactivity is detected in renal tubular epithelial cells with a membranous and cytoplasmic staining pattern. β-catenin shows characteristic linear membranous localization along the basolateral surfaces of tubular epithelium. The merged image demonstrates substantial co-localization along tubular epithelial cell membranes. (**b**) Low-grade ccRCC: PCDH9 is maintained in a subset of tumor cells with variable signal intensity and focal membranous accentuation. β-catenin shows mixed membranous and cytoplasmic positivity. The merged image reveals co-expression in tumor cell clusters retaining cohesive epithelial architecture. (**c**) High-grade ccRCC: PCDH9 expression is variable, with heterogeneous signal across the tumor parenchyma. β-catenin shows redistribution from membranous to predominantly cytoplasmic localization. The merged image demonstrates diminished and spatially altered co-localization compared to control tissue, with overlapping PCDH9 and β-catenin signals appearing as yellow fluorescence. Arrowheads indicate representative positive cells in each channel. Dashed boxes denote regions shown at higher magnification in the insets. Scale bar = 50 μm, represented by a yellow line.

These progressive architectural changes—from preserved tubular cohesion in G1 to discohesive cell nests with nuclear atypia in G4—provide the morphological substrate for the immunofluorescence reorganisation of PCDH9 and β-catenin (Figure 2) and the mesenchymal marker acquisition (examined in subsequent sections, and frame our central question of whether ccRCC progression reflects classical or partial EMT.

### 3.3. Immunofluorescence Expression of PCDH9 and CTNNB1 in Control and ccRCC Tissues

Double immunofluorescence staining was performed to evaluate the spatial expression and co-localization of PCDH9 (green) and β-catenin (CTNNB1; red) in control renal tissue and ccRCC tissues of varying histological grades.

In the control renal cortex, PCDH9 immunoreactivity was predominantly detected in the epithelial cells of renal tubules, exhibiting a membranous and cytoplasmic staining pattern in proximal and distal convoluted tubules, with additional positivity observed in glomerular structures (Figure 2a). β-catenin expression in control tissue was localized primarily to the basolateral membranes of tubular epithelial cells, with a characteristic linear membranous staining pattern. Additional immunoreactivity was detected in glomerular parietal epithelial cells. In merged images, PCDH9 and β-catenin signals showed substantial spatial overlap along the tubular epithelial cell membranes.

In low-grade ccRCC (Figure 2b), PCDH9 expression was maintained in a subset of tumor cells, displaying a cytoplasmic staining pattern with focal membranous accentuation. The signal intensity appeared variable across the tumor parenchyma, with some regions retaining moderate positivity while others showed reduced expression. β-catenin immunoreactivity in low-grade tumors showed a mixed pattern, with both membranous and cytoplasmic positivity detected in neoplastic cells. The merged images revealed areas of co-expression predominantly in tumor cell clusters retaining a cohesive epithelial architecture, suggesting preserved epithelial adhesion complex integrity in a proportion of low-grade tumor cells.

In high-grade ccRCC (Figure 2c), PCDH9 expression displayed a variable pattern, with areas of moderate cytoplasmic immunoreactivity interspersed with regions of reduced or absent signal. The heterogeneous staining pattern was consistent with the absence of statistically significant grade-dependent differences in PCDH9 protein expression. β-catenin immunoreactivity demonstrated a markedly heterogeneous pattern, with redistribution from the predominantly membranous localization observed in control tissue to a more diffuse cytoplasmic and, in some cells, perinuclear distribution. Focal loss of membranous β-catenin staining was observed in tumor cell nests exhibiting discohesive growth patterns. The merged images revealed diminished and spatially altered co-localization of PCDH9 and β-catenin compared to control tissue, with reduced overlap along cell-cell contact zones and increased cytoplasmic co-expression in individual tumor cells.

Pixel-level co-localization analysis (JACoP) on representative fields revealed a progressive decline in PCDH9/β-catenin spatial overlap from control to high-grade ccRCC. Pearson’s PCC averaged 0.61 ± 0.09 in CTRL, 0.43 ± 0.11 in low-grade, and 0.25 ± 0.13 in high-grade tumors. Manders’ M1 (fraction of PCDH9 signal co-localizing with β-catenin) decreased from 0.76 ± 0.07 in CTRL to 0.39 ± 0.14 in high-grade tumors, with a parallel decline in M2 (Supplementary Table S2). At the case level, Spear-man correlation between PCDH9 and β-catenin area-percentages was strong and significant in normal renal cortex (ρ = 0.654, *p* = 0.004, *n* = 17), consistent with the membranous co-localization observed qualitatively, but was completely lost in pooled ccRCC tissue (ρ = 0.181, *p* = 0.222, *n* = 47) and absent in both low-grade (ρ = 0.178, *p* = 0.330) and high-grade (ρ = 0.196, *p* = 0.483) subgroups (Supplementary Table S3). This protein-level loss of co-expression mirrors the loss of the PCDH9–CDH1 transcriptomic correlation in TCGA-KIRC (Section 3.5.2) and supports the selective disruption of epithelial adhesion co-regulation during ccRCC tumorigenesis.

**Figure 3:**
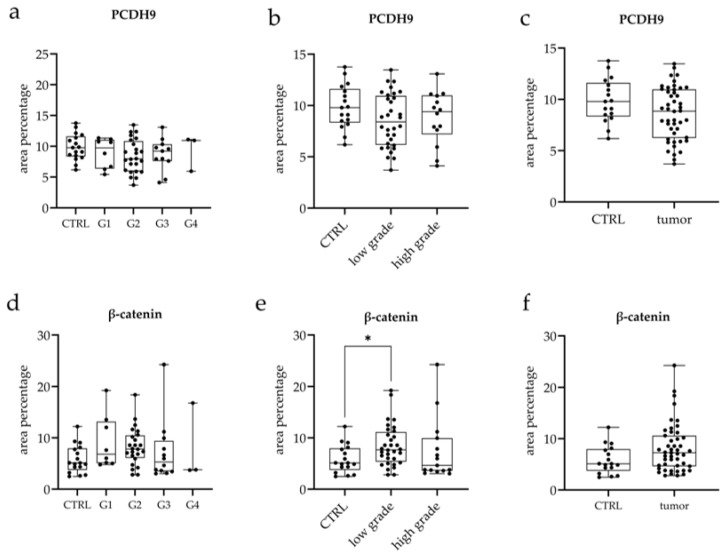
Quantitative analysis of PCDH9 (upper panels, (**a**–**c**)) and β-catenin (lower panels, (**d**–**f**)) immunoexpression in control renal cortex (CTRL) and clear cell renal cell carcinoma (ccRCC), expressed as area percentage of positive immunofluorescent signal. (**a**,**d**) Comparison across individual tumor grades (CTRL, G1, G2, G3, G4). For PCDH9 (**a**): one-way ANOVA with Tukey’s post-hoc; for β-catenin (**d**): Kruskal–Wallis test with Dunn’s post-hoc. (**b**,**e**) Comparison among CTRL, low-grade (G1 + G2), and high-grade (G3 + G4) groups. For PCDH9 (**b**): one-way ANOVA with Tukey’s post-hoc; for β-catenin (**e**): Kruskal–Wallis test with Dunn’s post-hoc. (**c**,**f**) Comparison between CTRL and pooled ccRCC tissue. For PCDH9 (**c**): Welch’s *t*-test (two-tailed); for β-catenin (**f**): Mann–Whitney U test (two-tailed). Data are presented as box-and-whisker plots (median, interquartile range, minimum–maximum) with individual data points for all analyzed samples. * *p* < 0.05.

Quantitative analysis of PCDH9 immunoexpression revealed no statistically significant difference between control tissue and pooled ccRCC tissue, although a strong trend toward lower expression in tumors was observed (*p* = 0.0583; Figure 3c). When stratified into control, low-grade, and high-grade groups, no significant overall difference was detected (*p* = 0.211; Figure 3b). Similarly, analysis across individual tumor grades showed no significant differences (*p* = 0.464; Figure 3a).

For β-catenin, comparison of control tissue versus all ccRCC samples combined revealed a trend toward higher expression in tumor tissue that did not reach statistical significance (Mann–Whitney U test, *p* = 0.073; Figure 3f). When stratified into three groups, a significant overall difference was detected (*p* = 0.045); post-hoc analysis revealed a statistically significant difference between CTRL and low-grade tumors (*p* = 0.0365; Figure 3e), while no significant difference was observed between CTRL and high-grade tumors. Analysis across individual tumor grades similarly showed no significant pairwise differences (Figure 3d).

### 3.4. Immunofluorescence Expression of SNAI1 and VIM in Control and ccRCC Tissues

**Figure 4:**
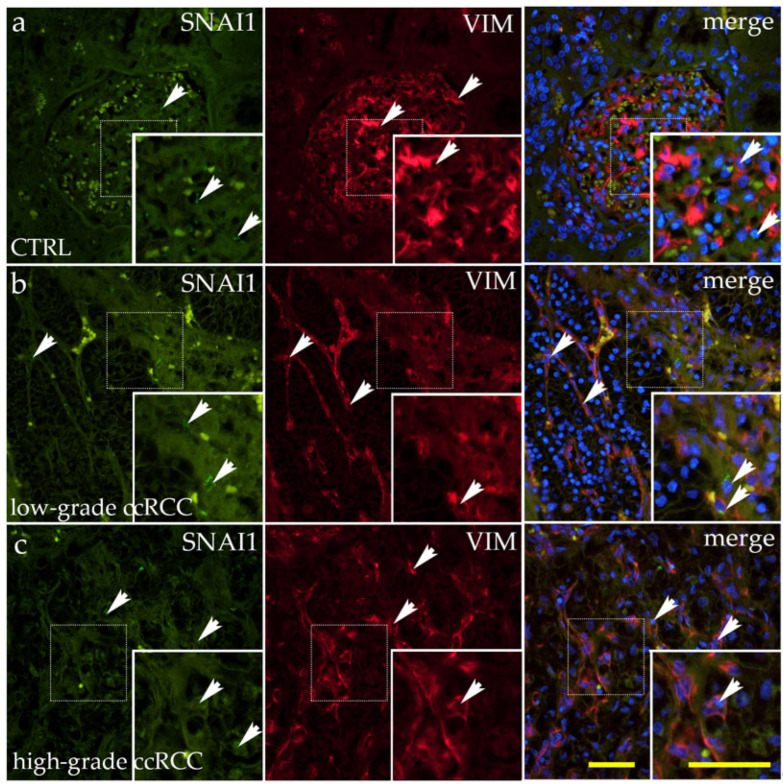
Representative double immunofluorescence micrographs showing the expression and co-localization of SNAI1 (green) and Vimentin (VIM; red) in control renal cortex and clear cell renal cell carcinoma (ccRCC). Nuclei were counterstained with DAPI (blue). (**a**) Control kidney tissue (CTRL): SNAI1 immunoreactivity is present in tubular epithelial cells, while VIM expression is restricted to the interstitial mesenchyme and glomerular structures. The merged image shows minimal co-localization, reflecting the distinct epithelial and mesenchymal compartments of normal renal parenchyma. (**b**) Low-grade ccRCC: SNAI1 appears as focal cytoplasmic positivity in tumor cells, and VIM is detected in both stromal cells and a subset of neoplastic cells. The merged image reveals areas of co-expression (yellow) in individual tumor cells, suggesting early epithelial–mesenchymal transition (EMT). (**c**) High-grade ccRCC: both SNAI1 and VIM are diffusely expressed throughout the tumor parenchyma and peritumoral stroma. The merged image demonstrates extensive co-localization (yellow), consistent with advanced EMT. Arrowheads indicate representative positive cells in each channel. Dashed boxes denote regions shown at higher magnification in the insets. Scale bar = 50 μm, represented by a yellow line.

Double immunofluorescence staining was performed to evaluate the spatial expression and co-localization of SNAI1 (green) and VIM (red) in control renal tissue and ccRCC tissues. In the control renal cortex, SNAI1 immunoreactivity was detected as a diffuse cytoplasmic signal within tubular epithelial cells, with moderate positivity observed in both proximal and distal convoluted tubules as well as in glomerular structures (arrowheads) (Figure 4a). VIM expression in control tissue was predominantly localized to the interstitial and perivascular mesenchymal compartments, with a characteristic filamentous cytoplasmic pattern in stromal cells. Additional immunoreactivity was observed within the glomerular tuft, likely corresponding to mesangial cells and podocytes (arrowheads). In merged images, SNAI1 (green) and VIM (red) signals showed largely distinct spatial distributions, with only limited overlap detected in peritubular regions, consistent with the predominantly epithelial phenotype of the normal renal parenchyma.

In low-grade ccRCC (Figure 4b), SNAI1 expression appeared as a focal, punctate cytoplasmic signal within individual tumor cells, with some positive cells scattered across the tumor parenchyma. VIM immunoreactivity was observed both in the surrounding stromal framework and, notably, in a subset of neoplastic cells that displayed elongated, fibroblast-like morphology, consistent with partial acquisition of mesenchymal characteristics. The merged images revealed areas of SNAI1 and VIM co-expression in individual tumor cells and along the tumor–stroma interface, indicative of cells undergoing early stages of EMT.

In high-grade ccRCC (Figure 4c), SNAI1 immunoreactivity was more diffusely distributed across the tumor parenchyma, with signal detected in the cytoplasm of numerous tumor cells as well as in the peritumoral stroma. VIM expression was markedly increased compared to both control tissue and low-grade ccRCC, displaying intense and widespread cytoplasmic positivity throughout the tumor mass and the adjacent stromal compartment. Merged images demonstrated extensive co-localization of SNAI1 and VIM across large areas of the tumor tissue, consistent with advanced EMT features in high-grade tumors. Compared to low-grade tumors, the co-expression pattern in high-grade ccRCC was more confluent and involved a larger proportion of the tumor cell population.

PCC values were low in control tissue (0.17 ± 0.08), reflecting the largely distinct epithelial (SNAI1) and interstitial-mesenchymal (VIM) compartments, and increased modestly in tumor fields (low-grade 0.31 ± 0.10; high-grade 0.41 ± 0.12; Supplementary Table S2). Manders’ coefficients were asymmetric: M1 (fraction of SNAI1 signal co-localizing with VIM) was consistently higher than M2 (fraction of VIM signal co-localizing with SNAI1), indicating that most SNAI1-positive tumor cells also expressed VIM, whereas much of the VIM-positive area belonged to SNAI1-negative stromal cells. At the case level, SNAI1 and VIM area-percentages showed no significant positive Spearman correlation in any group (CTRL: ρ = −0.366, *p* = 0.219; low-grade: ρ = −0.098, *p* = 0.595; high-grade: ρ = 0.200, *p* = 0.458; pooled ccRCC: ρ = −0.137, *p* = 0.352; Supplementary Table S3). Together, these findings indicate that SNAI1/VIM co-expression in ccRCC is focal and heterogeneous—confined to a subset of tumor cells rather than uniform and field-wide—consistent with the partial EMT model.

**Figure 5:**
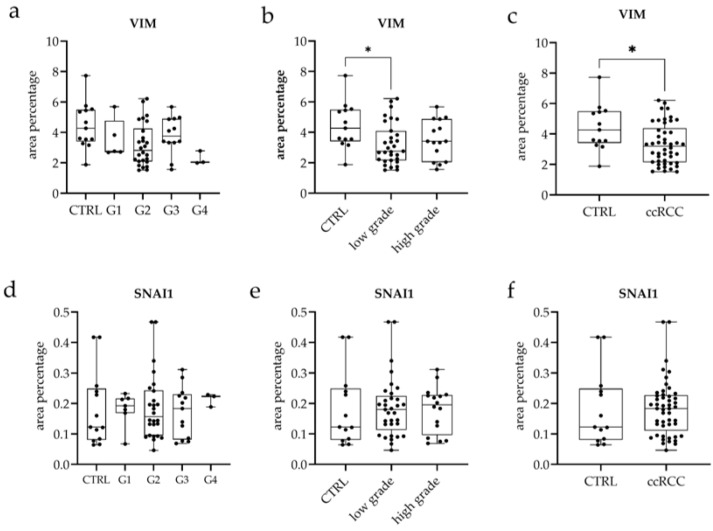
Quantitative analysis of Vimentin (VIM; upper panels) and SNAI1 (lower panels) immunoexpression in control renal cortex (CTRL) and clear cell renal cell carcinoma (ccRCC), expressed as area percentage of positive immunofluorescent signal. (**a**,**d**) Comparison across individual tumor grades (CTRL, G1, G2, G3, G4; Kruskal–Wallis test with Dunn’s post-hoc). (**b**,**e**) Comparison among CTRL, low-grade (G1 + G2), and high-grade (G3 + G4) groups (Kruskal–Wallis test with Dunn’s post-hoc). (**c**,**f**) Comparison between CTRL and pooled ccRCC tissue (Mann–Whitney U test, two-tailed). Data are presented as box-and-whisker plots (median, interquartile range, minimum–maximum) with individual data points for all analyzed samples. Asterisks indicate statistically significant differences (* *p* < 0.05).

No statistically significant difference in SNAI1 area percentage was observed between control tissue and pooled ccRCC tissue (*p* = 0.534; Figure 5c). Comparison among control, low-grade, and high-grade groups similarly showed no significant overall difference (*p* = 0.818; Figure 5b). When analyzed across all four individual grades, no significant differences were detected (*p* = 0.846; Figure 5a). SNAI1 immunoexpression remained relatively stable across all groups examined.

Comparison of control tissue versus all ccRCC samples combined revealed a significantly lower VIM area percentage in tumor tissue (*p* = 0.018; Figure 5f). When the cohort was stratified into control, low-grade, and high-grade groups, we detected a significant overall difference (*p* = 0.045). Analysis demonstrated significantly lower VIM expression in the low-grade group compared to controls (*p* = 0.047), while the difference between controls and the high-grade group did not reach statistical significance (*p* = 0.321; Figure 5e). Analysis across individual tumor grades revealed a similar pattern (*p* = 0.041), though no individual pairwise comparisons reached statistical significance (Figure 5d).

### 3.5. Bioinformatic Validation Using TCGA-KIRC Dataset

#### 3.5.1. *PCDH9* Is Downregulated While *VIM* Is Upregulated in ccRCC

**Figure 6:**
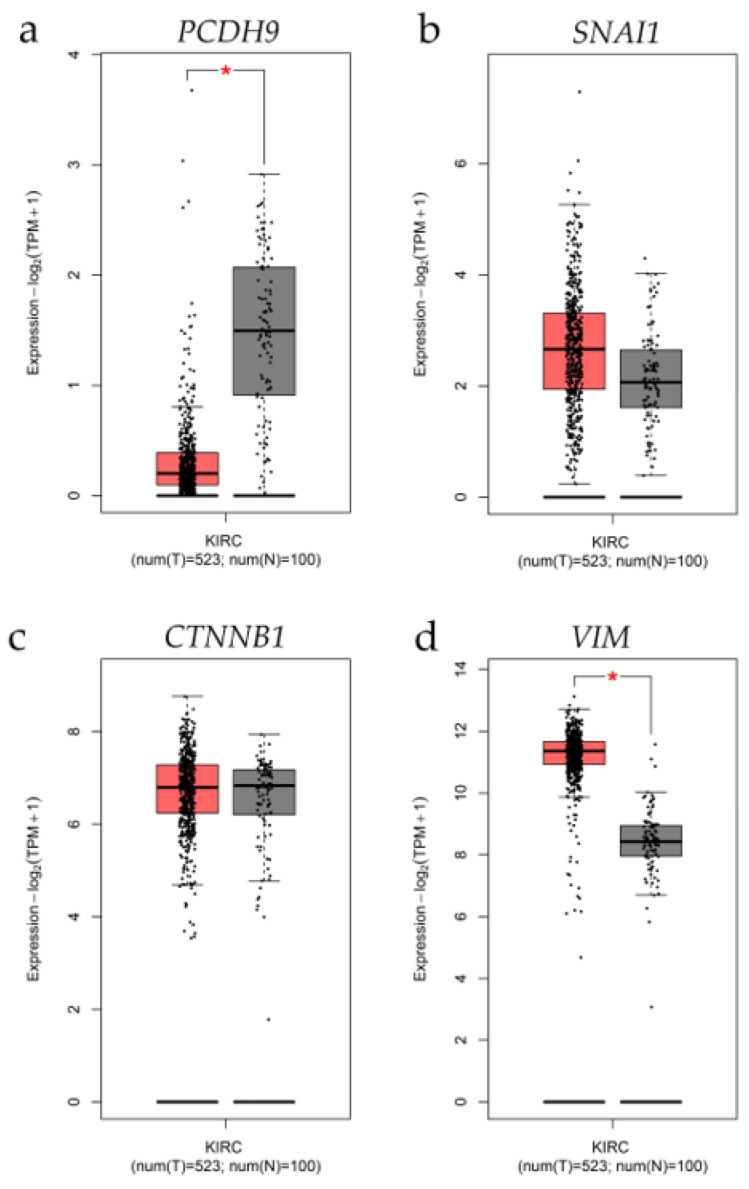
Differential expression of *PCDH9* and EMT-related genes in ccRCC. Box plots comparing gene expression levels between ccRCC tumor tissue (red, *n* = 523) and normal kidney tissue (grey, *n* = 100) from TCGA-KIRC and GTEx datasets. (**a**) *PCDH9* expression is significantly downregulated in ccRCC tumors compared to normal tissue (* *p* < 0.01). (**b**) *SNAI1* expression shows no statistically significant difference between tumor and normal tissue (|log_2_FC| < 1 or q ≥ 0.01). (**c**) *CTNNB1* (*β-catenin*) expression is not significantly different between tumor and normal tissue. (**d**) *VIM* (Vimentin) expression is significantly upregulated in ccRCC tumors (* *p* < 0.01). Expression values are presented as log_2_(TPM + 1). Statistical analysis was performed using one-way ANOVA with significance thresholds of |log_2_FC| ≥ 1 and q-value < 0.01. Asterisks (*) indicate statistically significant differences. Data were obtained from GEPIA2 (http://gepia2.cancer-pku.cn/, 27 February 2026).

To assess the expression status of *PCDH9* and EMT-related genes in ccRCC, we performed differential expression analysis comparing tumor (*n* = 523) and normal kidney tissue (*n* = 100) using the GEPIA2 platform (Figure 6).

*PCDH9* expression was significantly downregulated in ccRCC tumors compared to normal kidney tissue (*p* < 0.01; Figure 6a). The median expression of *PCDH9* in tumor samples was markedly lower, with the majority of tumor samples showing reduced expression levels compared to normal tissue. This finding supports the proposed tumor suppressor role of *PCDH9* in ccRCC.

In contrast, *VIM* (*Vimentin*), a canonical mesenchymal marker, showed significant upregulation in ccRCC tumors (*p* < 0.01; Figure 6d).

Interestingly, neither *SNAI1* nor *CTNNB1* showed statistically significant differential expression between tumor and normal tissue at the applied thresholds (|log_2_FC| ≥ 1, q < 0.01; Figure 6b,c). For *SNAI1*, although a trend toward higher expression in tumors was observed, the difference did not reach significance. *CTNNB1* mRNA levels were similar between tumor and normal tissue.

#### 3.5.2. *PCDH9* Expression Correlates with EMT Transcription Factors but Not with *E-Cadherin* in ccRCC

**Figure 7:**
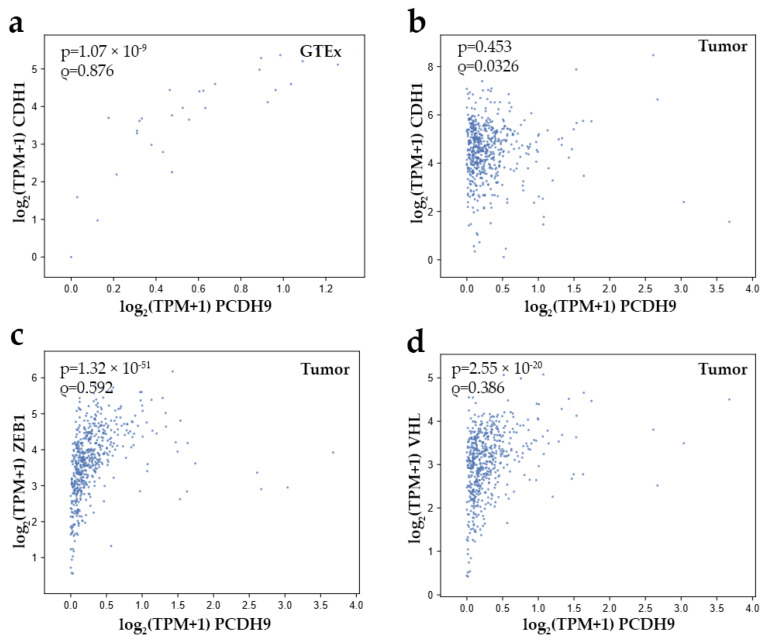
Correlation analysis of *PCDH9* expression with EMT markers, Wnt/β-catenin pathway components, and ccRCC-specific genes. (**a**) Scatter plot showing strong positive correlation between *PCDH9* and *CDH1* expression in normal kidney tissue (GTEx, *n* = 28; Spearman ρ = 0.876, *p* = 1.07 × 10^−9^). (**b**) Loss of *PCDH9*-*CDH1* correlation in ccRCC tumor tissue (TCGA-KIRC, *n* = 531; ρ = 0.033, *p* = 0.453). (**c**) Positive correlation between *PCDH9* and the EMT transcription factor *ZEB1* in ccRCC tumors (ρ = 0.592, *p* = 1.32 × 10^−51^). (**d**) Positive correlation between *PCDH9* and the tumor suppressor *VHL* in ccRCC tumors (ρ = 0.386, *p* = 2.55 × 10^−20^). Gene expression values are presented as log_2_(TPM + 1). Data were obtained from GEPIA3 (http://gepia3.bioinfoliu.com/, 27 February 2026).

To investigate the relationship between *PCDH9* and EMT-related genes in ccRCC, we performed correlation analysis using TCGA-KIRC tumor samples, peritumor tissue, and GTEx normal kidney data (Figure 7).

In normal kidney tissue (GTEx), *PCDH9* showed a strong positive correlation with *CDH1* (E-cadherin) (ρ = 0.876, *p* = 1.07 × 10^−9^; Figure 7a), consistent with their shared role in maintaining epithelial cell adhesion. This correlation was completely absent in ccRCC tumor samples (ρ = 0.033, *p* = 0.453; Figure 7b).

*PCDH9* expression in ccRCC tumors showed significant positive correlations with EMT-inducing transcription factors, including *ZEB1* (ρ = 0.592, *p* = 1.32 × 10^−51^; Figure 7c), *ZEB2* (ρ = 0.558, *p* = 8.52 × 10^−45^), *SNAI1* (ρ = 0.479, *p* = 7.71 × 10^−32^), and *SNAI2* (ρ = 0.526, *p* = 3.76 × 10^−39^) (Table S1).

Analysis of Wnt/β-catenin pathway components revealed positive correlations between *PCDH9* and *CTNNB1* (ρ = 0.560, *p* = 3.77 × 10^−45^), *AXIN2* (ρ = 0.496, *p* = 2.18 × 10^−34^), and *MYC* (ρ = 0.366, *p* = 2.54 × 10^−18^), suggesting that *PCDH9* does not function as an antagonist of β-catenin signaling in ccRCC (Table S1).

Furthermore, *PCDH9* demonstrated a moderate positive correlation with the ccRCC tumor suppressor *VHL* (ρ = 0.386, *p* = 2.55 × 10^−20^; Figure 7d) and *HIF1A* (ρ = 0.431, *p* = 1.91 × 10^−25^).

Control analyses confirmed weak but significant inverse correlations between *CDH1* and the mesenchymal markers *SNAI1* (ρ = −0.177, *p* = 4.07 × 10^−5^) and *VIM* (ρ = −0.135, *p* = 0.00176) in tumor tissue (Table S1).

#### 3.5.3. High *PCDH9* and *CTNNB1* Expression Is Associated with Favorable Prognosis

**Figure 8:**
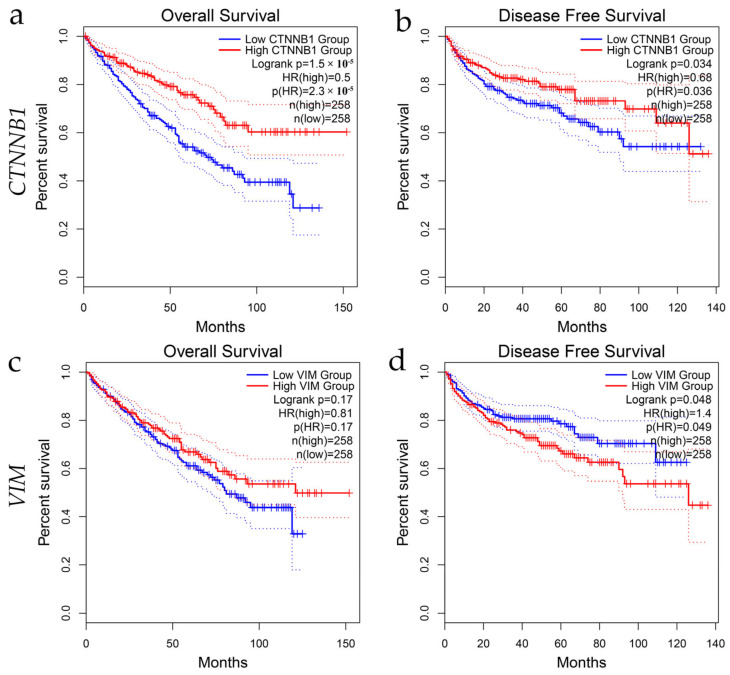
Kaplan–Meier survival analysis of PCDH9 and EMT-related genes in ccRCC. Overall survival (OS; left panels) and disease-free survival (DFS; right panels) curves for ccRCC patients stratified by median expression of CTNNB1 (**a**,**b**) and VIM (**c**,**d**). High expression groups are shown in red; low expression groups are shown in blue. Dotted lines represent 95% confidence intervals. (**a**) High CTNNB1 expression is strongly associated with improved OS (log-rank *p* = 1.5 × 10^−5^; HR = 0.5). (**b**) High CTNNB1 expression is associated with improved DFS (log-rank *p* = 0.034; HR = 0.68). (**c**) VIM expression shows no significant association with OS (log-rank *p* = 0.17; HR = 0.81). (**d**) High VIM expression is associated with worse DFS (log-rank *p* = 0.048; HR = 1.4). Sample sizes: *n*(high) = 256–258, *n*(low) = 252–258 per group. Data were obtained from GEPIA2 using the TCGA-KIRC dataset (http://gepia2.cancer-pku.cn/, 27 February 2026).

To evaluate the clinical relevance of *PCDH9* and EMT-related gene expression in ccRCC, we performed Kaplan–Meier survival analysis using TCGA-KIRC data (Figure 8).

High *PCDH9* expression was significantly associated with improved OS in ccRCC patients (*p* = 0.024; Figure S1a). Patients in the high *PCDH9* expression group demonstrated approximately 30% reduced risk of death compared to those with low *PCDH9* expression. However, *PCDH9* expression was not significantly associated with DFS (*p* = 0.83; Figure S1b).

*CTNNB1* (*β-catenin*) showed the strongest prognostic significance among the analyzed genes. High *CTNNB1* expression was associated with markedly improved OS (*p* = 2.3 × 10^−5^; Figure 8a) and DFS (*p* = 0.034; Figure 8b). Patients with high *CTNNB1* expression had approximately 50% reduced risk of death and 32% reduced risk of disease recurrence compared to the low expression group.

In contrast, *SNAI1* expression showed no significant association with either OS (*p* = 0.094; Figure S1c) or DFS (*p* = 0.53; Figure S1d), although a trend toward worse OS in the high *SNAI1* group was observed.

For *VIM*, no significant association with OS was detected (*p* = 0.17; Figure 8c). However, high *VIM* expression was significantly associated with worse DFS (*p* = 0.048; Figure 8d), indicating that elevated *VIM* expression may predict earlier disease recurrence in ccRCC patients.

Collectively, these findings support a tumor-suppressive role for *PCDH9* in ccRCC and highlight the prognostic value of *CTNNB1* expression. The association between high *VIM* expression and reduced DFS is consistent with *VIM’s* role as a mesenchymal marker associated with tumor progression.

#### 3.5.4. Multivariate Analysis Reveals Endpoint-Specific Prognostic Signatures

**Figure 9:**
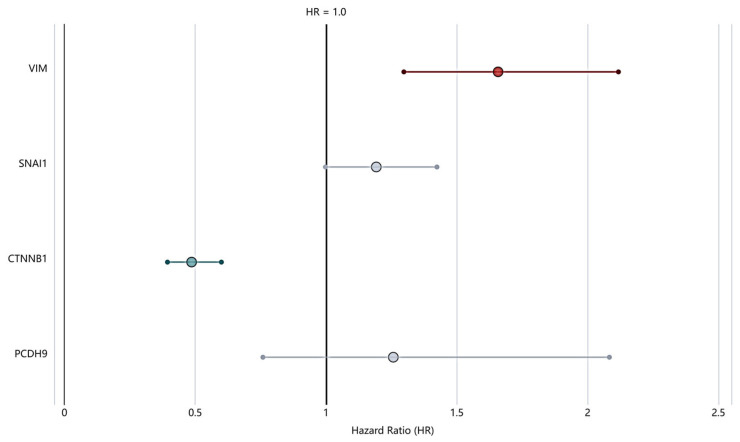
Multivariate Cox regression analysis of *PCDH9* and EMT-related genes in ccRCC. Forest plots displaying hazard ratios (HR) and 95% confidence intervals (CI) from multivariate Cox proportional hazards regression analysis. All four genes (*VIM*, *SNAI1*, *CTNNB1*, *PCDH9*) were included simultaneously in the progression-free interval (PFI model): *CTNNB1* is significantly associated with reduced risk of progression (HR = 0.486, 95% CI: 0.394–0.600, *p* = 1.88 × 10^−11^), while *VIM* is significantly associated with increased risk of progression (HR = 1.658, 95% CI: 1.298–2.118, *p* = 5.24 × 10^−5^). *SNAI1* shows a trend toward a worse outcome (HR = 1.192, *p* = 0.0526). PCDH9 is not significant (HR = 1.257, *p* = 0.374). Points with confidence intervals entirely to the left indicate a protective effect (teal); points entirely to the right indicate increased risk (red); overlapping HR = 1.0 indicates non-significance (grey). Data were obtained from GEPIA3 using the TCGA-KIRC dataset (https://gepia3.bioinfoliu.com/, 27 February 2026).

Multivariate Cox regression analysis was performed to identify independent prognostic factors, using both progression-free interval (PFI) and overall survival (OS) as endpoints (Figure 9).

In the PFI model, two genes demonstrated independent prognostic significance (Figure 9). *CTNNB1* was strongly associated with reduced risk of disease progression (HR = 0.486, 95% CI: 0.394–0.600, *p* = 1.88 × 10^−11^), indicating that high *CTNNB1* expression confers approximately 51% reduced risk of progression. Notably, *VIM* emerged as a significant independent predictor of disease progression (HR = 1.658, 95% CI: 1.298–2.118, *p* = 5.24 × 10^−5^), with high *VIM* expression associated with 66% increased risk of progression. *SNAI1* showed a trend toward a worse outcome but did not reach statistical significance (HR = 1.192, 95% CI: 0.998–1.424, *p* = 0.0526). *PCDH9* was not independently associated with PFI (HR = 1.257, 95% CI: 0.759–2.083, *p* = 0.374).

The OS model revealed a different prognostic pattern. *CTNNB1* remained the strongest protective factor (HR = 0.494, 95% CI: 0.405–0.603, *p* = 4.71 × 10^−12^). However, in contrast to the PFI analysis, *SNAI1* was significantly associated with increased mortality risk (HR = 1.347, 95% CI: 1.135–1.598, *p* = 0.00065), while *VIM* showed only a non-significant trend (HR = 1.186, 95% CI: 0.978–1.440, *p* = 0.0835). *PCDH9* was not independently associated with OS (HR = 1.214, 95% CI: 0.782–1.884, *p* = 0.387) (Figure S1).

## 4. Discussion

This study provides a comprehensive, multi-level analysis of PCDH9 and EMT-related markers in ccRCC, integrating tissue-level immunofluorescence findings with transcriptomic validation using the TCGA-KIRC dataset. Our results reveal several novel insights into the molecular landscape of EMT in ccRCC and identify clinically relevant prognostic signatures that may inform patient stratification.

Our analysis demonstrates significant *PCDH9* downregulation at the mRNA level in ccRCC, establishing *PCDH9* as a consistently suppressed gene in this malignancy. This finding extends previous observations in hepatocellular carcinoma and glioblastoma, where *PCDH9* loss promotes tumor cell migration and invasion by activating GSK-3β and modulating the Wnt/β-catenin pathway [11,12,32].

The significant positive correlation between *PCDH9* and *VHL* expression suggests that PCDH9 suppression may be mechanistically linked to *VHL* inactivation, which occurs in approximately 90% of sporadic ccRCC cases [21,22]. *PCDH9* loss may result from hypoxia-driven transcriptional repression or epigenetic silencing through promoter hypermethylation, as reported in glioblastoma [12].

The positive correlation between *PCDH9* and *HIF1A* mRNA, though seemingly paradoxical, may reflect the well-established fact that HIF-1α activity is predominantly regulated at the post-translational level through oxygen-dependent VHL-mediated proteasomal degradation, rather than at the level of *HIF1A* transcription [21].

Perhaps the most striking finding from our correlation analysis is the complete loss of correlation between *PCDH9* and *CDH1* in tumor tissue compared with normal kidney tissue. In healthy renal epithelium, these adhesion molecules appear to be co-regulated, potentially through shared transcriptional programs. Epithelial-specific transcription factors such as *GRHL2* and *OVOL1/2*, which are known to coordinate the expression of multiple adhesion molecules [33,34], represent plausible candidates for mediating this co-regulation, although direct evidence for their role in *PCDH9* transcription is currently lacking. In line with current EMT models that describe epithelial identity as a modular and partially reversible transcriptional program [35], the observed uncoupling between *PCDH9* and *CDH1* expression in ccRCC may reflect selective reorganization of epithelial transcriptional networks rather than a complete loss of epithelial phenotype.

Notably, at the protein level, PCDH9 immunoexpression showed a strong trend toward reduction in ccRCC tissue compared to normal renal cortex (*p* = 0.058), which, while not reaching the conventional threshold for statistical significance, is consistent with the transcriptomic findings and likely reflects limited statistical power due to the modest sample size (*n* = 48) and inherent variability of area-based quantification. Given that the TCGA-KIRC dataset, with a substantially larger sample size (*n* > 500), confirmed significant *PCDH9* downregulation, the tissue-level trend should not be disregarded but rather interpreted as convergent evidence supporting *PCDH9* suppression in ccRCC.

The univariate prognostic significance of *PCDH9* was lost in multivariate analysis, most likely due to its strong correlation with *CTNNB1*. Experimental evidence indicates that *PCDH9* suppresses EMT through activation of GSK-3β, leading to reduced β-catenin stability and downstream EMT transcriptional activity [32]. Regardless, the coordinate assessment of *PCDH9* and *CTNNB1* may provide a more complete picture of epithelial adhesion status than either marker alone.

The unexpected positive correlations between *PCDH9* and EMT transcription factors (*ZEB1*, *ZEB2*, *SNAI1*, and *SNAI2*) challenge the classical EMT model, which predicts inverse relationships between epithelial adhesion molecules and EMT-inducing transcription factors. These findings align with the emerging concept of partial or hybrid EMT, in which cells co-express both epithelial and mesenchymal markers to varying degrees rather than undergoing a complete phenotypic switch [35,36,37]. Several lines of evidence from our study support the notion that ccRCC exhibits a partial EMT phenotype. First, the positive correlations between *PCDH9* and EMT-inducing transcription factors (EMT-TFs) suggest that tumors with relatively preserved epithelial features also express higher levels of EMT-TFs—a pattern incompatible with complete EMT, where these gene classes are mutually exclusive. Second, the weak inverse correlations between *CDH1* and mesenchymal markers (*CDH1*–*SNAI1*; *CDH1*–*VIM*) contrast with the strong inverse correlations reported in cancers undergoing classical EMT, such as claudin-low breast cancer [38,39,40]. Third, the independent prognostic significance of both epithelial (*CTNNB1*) and mesenchymal (*VIM*) markers in multivariate analysis indicates that both components of the epithelial–mesenchymal spectrum contribute to clinical outcomes—a pattern not expected in tumors with complete EMT.

Recent single-cell RNA sequencing studies directly corroborate our findings. Lombardi et al. identified three conserved transcriptional modules in ccRCC—a differentiated proximal-tubule-like state, an EMT-like state, and a cell-injury state—existing as a continuum rather than discrete populations [41]. Similarly, Zvirblyte et al. discovered a tumor-associated endothelial subpopulation enriched for EMT pathway genes and associated with poor survival in TCGA-KIRC, further demonstrating the pervasive nature of partial EMT in the ccRCC microenvironment [42]. An additional consideration specific to ccRCC is that proximal tubular epithelial cells—the cells of origin—can co-express epithelial markers and Vimentin even under injury or stress [43,44], blurring the classical EMT boundary and potentially explaining why this malignancy exhibits partial rather than complete EMT [10,45,46].

*CTNNB1* emerged as the most robust prognostic factor, consistently associated with favorable outcomes in both univariate and multivariate analyses. This finding reflects the dual role of β-catenin in cancer biology. While nuclear *β-catenin* acts as an oncogenic transcriptional co-activator driving *MYC* and *CCND1* expression [15,16], it also serves an essential structural function at adherens junctions, linking E-cadherin to the actin cytoskeleton [47,48,49].

In ccRCC, the protective association of high *CTNNB1* expression likely reflects its structural rather than transcriptional role. This interpretation is supported by the strong positive correlation with *PCDH9*, suggesting co-regulation within an epithelial gene program, the absence of *CTNNB1* differential expression between tumor and normal tissue, arguing against Wnt pathway activation, and the consistent protective effect across survival endpoints, which would not be expected for an oncogenic driver. These findings align with reports that preserved membranous β-catenin immunostaining is associated with lower tumor stage and improved survival in RCC [50]. An important caveat is that *CTNNB1* mRNA expression may not fully capture β-catenin subcellular localization, and future studies incorporating compartment-specific immunohistochemical assessment would provide additional mechanistic insight.

One of the most clinically significant findings is the identification of endpoint-specific prognostic signatures. For progression-free interval, *VIM* was the dominant adverse factor, while for overall survival, *SNAI1* was significantly adverse and *VIM* showed only a trend. This divergence has coherent biological underpinnings. It should be noted that the univariate analysis employed disease-free survival (DFS) as defined by GEPIA2, while the multivariate analysis used progression-free interval (PFI) as available through GEPIA3. Although these endpoints are conceptually related, they are not identical—DFS includes death as an event, whereas PFI captures disease progression specifically. Despite this methodological distinction, the consistent directionality of *VIM*’s adverse association across both endpoints strengthens the biological interpretation.

Vimentin, as a structural component of the mesenchymal cytoskeleton, directly enables the cellular plasticity required for invasion and metastasis [17,19,51]. Its association with disease progression is mechanistically coherent: tumors with higher VIM content possess the structural machinery for invasion. In contrast, *SNAI1* orchestrates broad transcriptional programs beyond direct EMT regulation, including apoptosis resistance, cancer stem cell maintenance, metabolic reprogramming, and immune evasion [9,52,53]. Its selective association with overall survival suggests influence on mortality through mechanisms extending beyond metastasis, potentially including resistance to tyrosine kinase inhibitors and immune checkpoint inhibitors—the current mainstays of advanced ccRCC treatment [18,52]. These endpoint-specific roles have practical implications: *VIM* expression may identify patients at risk of early progression who could benefit from intensified surveillance, while *SNAI1* expression could identify patients at risk of poor long-term survival who might be prioritized for novel therapeutic approaches. A combined *CTNNB1*/*VIM*/*SNAI1* prognostic panel could stratify ccRCC patients more effectively than any single marker and warrants prospective validation.

The most notable discordance of our study concerns *VIM*. TCGA-KIRC showed significant mRNA upregulation in tumors, while our immunofluorescence revealed higher VIM area percentage in control tissue. The fundamental distinction between bulk tissue immunofluorescence quantification and transcriptomic analysis may explain this [54,55]. Our approach measured the total area of the positive signal without cell-type distinction. In the normal renal cortex, Vimentin is abundantly expressed by interstitial fibroblasts, pericytes, mesangial cells, and podocytes, which collectively occupy a substantial tissue area. In ccRCC, although tumor cells acquire de novo Vimentin through EMT, the total tissue-level signal may be lower due to loss of the vimentin-rich normal interstitium. TCGA RNA-seq, by contrast, reflects aggregate transcriptional output, in which tumor cell–derived VIM mRNA predominates due to high tumor cell content. Similarly, *PCDH9* showed mRNA downregulation but no significant protein-level difference, possibly reflecting post-transcriptional regulation or limited sensitivity of area-based quantification. For *SNAI1*, both approaches showed no differential expression—concordant with its predominantly post-translational regulation. These findings underscore that tissue-level immunofluorescence and transcriptomic analyses provide complementary rather than interchangeable information, and highlight the need for cell-type-resolved methods such as spatial transcriptomics or multiplex digital pathology in future studies.

Several limitations of the study should be acknowledged. The retrospective design and modest sample size—particularly for G1 (*n* = 7) and G4 (*n* = 3) tumors—may limit generalizability, though the grade distribution reflects clinical practice. Importantly, not all patients had sufficient normal cortical tissue for paired-control analysis, resulting in variable control group sizes across staining panels (*n* = 13–17). We could not correlate tissue-level protein expression with patient survival due to a lack of long-term follow-up; prognostic associations derive from TCGA data and require independent validation. TCGA-KIRC lacks detailed treatment information, and the therapeutic landscape has changed substantially since most TCGA samples were collected. Area-percentage quantification, when applied to bulk tissue fields, does not distinguish between tumor parenchyma, stromal cells, and residual normal structures, which may obscure cell-type-specific changes—particularly for VIM. Finally, bulk analyses cannot capture intratumoral heterogeneity, and our study lacks functional validation of the causal relationships between marker expression and outcomes.

## 5. Conclusions

This study demonstrates that ccRCC is characterized by significant downregulation of *PCDH9 mRNA* and complete disruption of the *PCDH9-CDH1* co-regulatory relationship observed in normal kidney, supporting a tumor-suppressive role for *PCDH9* in this malignancy. The unexpected positive correlations between *PCDH9* and EMT transcription factors, combined with weak inverse correlations between *CDH1* and mesenchymal markers, indicate that ccRCC exhibits a partial EMT phenotype rather than classical EMT. Multivariate survival analysis identified endpoint-specific prognostic signatures, with *CTNNB1* consistently protective, *VIM* independently predicting disease progression, and *SNAI1* predicting overall mortality. The discordance between tissue-level immunofluorescence and transcriptomic data highlights the influence of cellular heterogeneity on bulk quantification and underscores the need for cell-type-resolved analyses in future studies. The partial discordance between tissue-level immunofluorescence and transcriptomic data—most notably for VIM—underscores that these two analytical levels capture complementary biological information, shaped by the distinct cellular composition of the tissue compartments examined.

A combined *CTNNB1*/*VIM*/*SNAI1* prognostic panel warrants prospective validation for risk stratification of ccRCC patients.

## Figures and Tables

**Table 1:** Primary and secondary antibodies used for immunofluorescence staining.

Antibodies		Catalog Number	Host	Dilution	Source
Primary	PCDH9 Polyclonal antibody	25090-1-AP	Rabbit	1:200	Proteintech Group, Inc. (Rosemont, IL, USA)
	beta-Catenin (L54E2) Mouse Monoclonal Antibody #2677	CST 2677	Mouse	1:200	Cell Signaling Technology, Inc. (CST) Danvers, MA, USA
	Snail (L70G2) Mouse Monoclonal Antibody	CST 3895	Mouse	1:200	
	Vimentin (D21H3) Rabbit Monoclonal Antibody	CST 5741	Rabbit	1:200	
Secondary	Alexa Fluor^®^ 488 Affini-Pure^®^ Donkey Anti-Rabbit IgG (H + L)	711-545-152	Donkey	1:400	Jackson Immuno Research Laboratories, Inc., West Grove, PA, USA
	Alexa Fluor^®^ 488 AffiniPure™ Donkey Anti-Mouse IgG (H + L)	715-545-150	Donkey	1:400	
	Rhodamine Red™-X (RRX) AffiniPure™ Donkey Anti-Mouse IgG (H + L)	715-295-151	Donkey	1:400	
	Rhodamine RedTM-X (RRX) AffiniPureTM Donkey Anti-Rabbit IgG (H + L)	711-295-152	Donkey	1:400	

**Table 2:** Clinical and pathological characteristics of the study cohort.

Variable	Total (*n* = 48)	G1 (*n* = 7)	G2 (*n* = 24)	G3 (*n* = 14)	G4 (*n* = 3)	*p*-Value
Age (years)						
Mean ± SD	68.7 ± 11.3	67.0 ± 8.4	67.1 ± 11.6	71.5 ± 12.1	72.7 ± 13.2	0.545 ᵃ
Median (range)	72 (39–87)	70 (54–76)	72 (39–81)	75 (43–87)	70 (61–87)	
Sex, *n* (%)						0.989 ᵇ
Male	33 (68.8%)	5 (71.4%)	16 (66.7%)	10 (71.4%)	2 (66.7%)	
Female	15 (31.2%)	2 (28.6%)	8 (33.3%)	4 (28.6%)	1 (33.3%)	
Tumor size (cm)						
Mean ± SD	5.9 ± 3.5	4.0 ± 2.3	4.7 ± 1.9	7.8 ± 4.6	11.0 ± 2.0	**0.009 ᵃ**
Laterality, *n* (%)						0.504 ᵇ
Right	23 (50.0%)	3 (42.9%)	9 (40.9%)	9 (64.3%)	2 (66.7%)	
Left	23 (50.0%)	4 (57.1%)	13 (59.1%)	5 (35.7%)	1 (33.3%)	
pT stage, *n* (%)						**—** ^c^
pT1 (pT1a + pT1b)	26 (54.2%)	6 (85.7%)	15 (62.5%)	5 (35.7%)	0 (0.0%)	
pT2 (pT2a + pT2b)	2 (4.2%)	0 (0.0%)	1 (4.2%)	1 (7.1%)	0 (0.0%)	
pT3a	20 (41.7%)	1 (14.3%)	8 (33.3%)	8 (57.1%)	3 (100.0%)	
Focality, *n* (%)						**—** ^c^
Unifocal	45 (93.8%)	7 (100%)	24 (100%)	11 (78.6%)	3 (100%)	
Multifocal	3 (6.2%)	0 (0%)	0 (0%)	3 (21.4%)	0 (0%)	

ᵃ Kruskal–Wallis test; ᵇ Chi-square test. ^c^ Not tested; expected cell frequencies were too low for valid chi-square analysis due to small subgroup sizes. Bold indicates statistical significance (*p* < 0.05). Laterality percentages for G2 are based on 22 patients with available data (2 cases with unrecorded laterality).

**Table 3:** Comparison of low-grade vs. high-grade ccRCC.

Variable	Low-Grade (G1 + G2, *n* = 31)	High-Grade (G3 + G4, *n* = 17)	*p*-Value
Age (years), mean ± SD	67.1 ± 10.8	71.7 ± 11.9	0.164 ^c^
Sex, Male/Female	21/10	12/5	1.000 ᵈ
Tumor size (cm), mean ± SD	4.5 ± 2.0	8.3 ± 4.4	**0.004 ^c^**

^c^ Mann–Whitney U test; ᵈ Fisher’s exact test. Bold indicates statistical significance (*p* < 0.05).

## Data Availability

The original contributions presented in this study are included in the article. Further inquiries can be directed to the corresponding author. Gene expression and survival data for *PCDH9*, *CTNNB1*, *VIM*, and *SNAI1* in clear cell renal cell carcinoma (KIRC) were obtained from publicly accessible databases, including the Gene Expression Profiling Interactive Analysis tools GEPIA2 (http://gepia2.cancer-pku.cn/, 27 February 2026) and GEPIA3 (https://gepia3.bioinfoliu.com/, 27 February 2026). All resources were accessed on 30 January 2026. These databases provide transcriptomic and clinical data that support the differential expression and survival analyses presented in this study.

## Supplementary Materials

The following supporting information can be downloaded at: https://www.mdpi.com/article/10.3390/jpm16060279/s1 Table S1. Spearman correlation analysis of *PCDH9* expression with EMT markers, Table S2. Pixel-level co-localization analysis of PCDH9/β-catenin and SNAI1/VIM in control renal cortex and ccRCC, assessed by JACoP (ImageJ) with Costes’ automatic thresholding. Table S3. Per-case Spearman correlation between paired marker area-percentages within each group. Figure S1. Kaplan-Meier survival analysis of PCDH9 and EMT-related genes in ccRCC. Figure S2. Multivariate Cox regression analysis of PCDH9 and EMT-related genes in ccRCC—overall survival (OS) model.

## Abbreviations

The following abbreviations are used in this manuscript:

ANOVA	analysis of variance
CA9	carbonic anhydrase 9
ccRCC	clear cell renal cell carcinoma
CDH1	cadherin 1 (E-cadherin)
CDH2	cadherin 2 (N-cadherin)
CI	confidence interval
CTNNB1	catenin beta 1 (β-catenin)
DAPI	4′,6-diamidino-2-phenylindole
DFS	disease-free survival
EMT	epithelial–mesenchymal transition
EMT-TF	EMT-inducing transcription factor
FFPE	formalin-fixed, paraffin-embedded
GEPIA	Gene Expression Profiling Interactive Analysis
GTEx	Genotype-Tissue Expression
H&E	hematoxylin and eosin
HIF1A	hypoxia-inducible factor 1 subunit alpha
HR	hazard ratio
KIRC	kidney renal clear cell carcinoma
OS	overall survival
PBS	phosphate-buffered saline
PCDH9	protocadherin 9
PFA	paraformaldehyde
PFI	progression-free interval
SD	standard deviation
SNAI1	snail family transcriptional repressor 1
SNAI2	snail family transcriptional repressor 2
TCGA	The Cancer Genome Atlas
TPM	transcripts per million
VHL	von Hippel–Lindau
VIM	vimentin
WHO/ISUP	World Health Organization/International Society of Urological Pathology
ZEB1	zinc finger E-box binding homeobox 1
ZEB2	zinc finger E-box binding homeobox 2
