# Supplementary material for: Epithelial–Mesenchymal Transition Markers in Clear Cell Renal Cell Carcinoma: Expression Patterns and Prognostic Significance
Source: J Pers Med. 2026 May 24;16(6):279. doi: 10.3390/jpm16060279 (PMC13302423; doi:10.3390/jpm16060279)
Supplement: Supplementary file 1 [file jpm-16-00279-s001.zip › jpm-4204447-supplementary.pdf]

Journal of Personalized Medicine

Supplementary Material

Table S1. Spearman correlation analysis of *PCDH9* expression with EMT markers, Wnt/β-catenin pathway components, and ccRCC-specific genes across three tissue types. Summary table of Spearman rank correlation coefficients (ϱ) and corresponding p-values for *PCDH9* paired with selected genes across KIRC tumor tissue (n = 531), KIRC peritumor tissue (n = 72), and GTEx normal kidney tissue (n = 28). Gene pairs are organized by functional category: EMT markers (*CDH1, SNAI1, SNAI2, VIM, ZEB1, ZEB2, TWIST1, CDH2*), Wnt/β-catenin signaling pathway components (*CTNNB1, AXIN2, MYC, CCND1, LEF1*), ccRCC-specific markers (*VHL, HIF1A, CA9*), and control pairs (*CDH1–SNAI1, CDH1–VIM*). Gene expression values were log^2-transformed (TPM + 1) prior to analysis. Data were obtained from GEPIA3 (http://gepia3.bioinfoliu.com/).

Gene pairs		KIRC TUMOR (n=531)		KIRC PERITUMOR (n=72)		GTEx KIDNEY (n=28)	
Gene A	Gene B	Spearman R	p	Spearman R	p	Spearman R	p
EMT markers							
PCDH9	*CDH1*	0,0326	0,453	0,556	4,05E-07	0,876	1,07E-09
PCDH9	*SNAI1*	0,479	7,71E-32	-0,0687	0,566	0,568	0,00163
PCDH9	*SNAI2*	0,526	3,76E-39	-0,0615	0,608	0,717	1,77E-05
PCDH9	*VIM*	0,242	1,74E-08	-0,143	0,23	0,666	0,000109
PCDH9	*ZEB1*	0,592	1,32E-51	0,106	0,377	0,732	9,46E-06
PCDH9	*ZEB2*	0,558	8,52E-45	-6,43E-05	1	0,669	9,92E-05
PCDH9	*TWIST1*	0,4	8,28E-22	-0,181	0,127	0,243	0,213
PCDH9	*CDH2*	0,233	5,90E-08	-0,272	0,0209	0,329	0,0873
Wnt/β-CATENIN signalling pathway							
PCDH9	*CTNNB1*	0,56	3,77E-45	0,421	0,000232	0,805	2,47E-07
PCDH9	*AXIN2*	0,496	2,18E-34	0,31	0,00803	0,806	2,24E-07
PCDH9	*MYC*	0,366	2,54E-18	-0,0476	0,692	0,464	0,0129
PCDH9	*CCND1*	0,279	6,13E-11	0,501	7,45E-06	0,601	0,000716
PCDH9	*LEF1*	0,107	0,014	0,182	0,126	0,415	0,0282
ccRCC - specific markers							
PCDH9	*VHL*	0,386	2,55E-20	0,524	2,28E-06	0,809	1,92E-07
PCDH9	*HIF1A*	0,431	1,91E-25	0,313	0,00744	0,586	0,00106
PCDH9	*CA9*	0,0406	0,351	-0,0377	0,753	0,296	0,126
controls							
CDH1	*SNAI1*	-0,177	4,07E-05	0,0223	0,853	0,635	0,000287
CDH1	*VIM*	-0,135	0,00176	0,0501	0,676	0,784	7,92E-07

Table S2. Pixel-level co-localization analysis of PCDH9/β-catenin and SNAI1/VIM in control renal cortex and ccRCC, assessed by JACoP (ImageJ) with Costes' automatic thresholding.

A. PCDH9 / β-catenin					
Group	n (fields)	PCC (mean ± SD)	M1 (mean ± SD)	M2 (mean ± SD)	Costes' P
CTRL	5	0.61 ± 0.09	0.76 ± 0.07	0.72 ± 0.08	> 0.95
Low-grade	5	0.43 ± 0.11	0.60 ± 0.10	0.56 ± 0.12	> 0.95
High-grade	5	0.25 ± 0.13	0.39 ± 0.14	0.35 ± 0.15	> 0.95
B. SNAI1 / Vimentin					
CTRL	5	0.17 ± 0.08	0.43 ± 0.12	0.21 ± 0.09	0.78–0.95
Low-grade	5	0.31 ± 0.10	0.54 ± 0.11	0.30 ± 0.12	> 0.95
High-grade	5	0.41 ± 0.12	0.66 ± 0.11	0.43 ± 0.13	> 0.95

PCC = Pearson's correlation coefficient. M1 = Manders' overlap coefficient (fraction of green-channel signal co-localizing with red-channel signal). M2 = fraction of red-channel signal co-localizing with green-channel signal. Costes' automatic thresholding was applied independently to each channel; 200-iteration Costes' randomisation was used to assess statistical significance of co-localization ($P > 0.95$ indicates co-localization significantly above chance). Five representative double-stained fields per group were analysed at ×40 magnification. Green channel: PCDH9 (panel A) or SNAI1 (panel B); red channel: β-catenin (panel A) or VIM (panel B).

Table S3. Per-case Spearman correlation between paired marker area-percentages within each group.

A. PCDH9 vs β-catenin				
Group	n	Spearman ρ	p-value	Interpretation
CTRL	17	0.654	0.004	Strong positive — co-regulated in normal epithelium
Low-grade ccRCC	32	0.178	0.330	No significant co-regulation
High-grade ccRCC	15	0.196	0.483	No significant co-regulation
All ccRCC pooled	47	0.181	0.222	Co-regulation completely lost in tumor
B. SNAI1 vs Vimentin				
CTRL	13	−0.366	0.219	No positive correlation (distinct compartments)
Low-grade ccRCC	32	−0.098	0.595	No correlation
High-grade ccRCC	16	0.200	0.458	Weak trend toward positive (focal co-expression)
All ccRCC pooled	48	−0.137	0.352	No correlation — co-expression is focal, not field-wide

Spearman's rank correlation coefficient (ρ) was computed between per-case area-percentages of paired markers (PCDH9 vs β-catenin; SNAI1 vs Vimentin) within each group. Area-percentages were obtained by immunofluorescence quantification (ImageJ, median filter subtraction and color thresholding) on 10 non-overlapping fields per case at ×40 magnification. Where channel sample sizes differed slightly within a group (e.g., SNAI1 G1 $n = 7$ vs. VIM G1 $n = 6$), the smaller n was used. Statistical significance set at $p < 0.05$.

https://doi.org/10.3390/jpm16060279

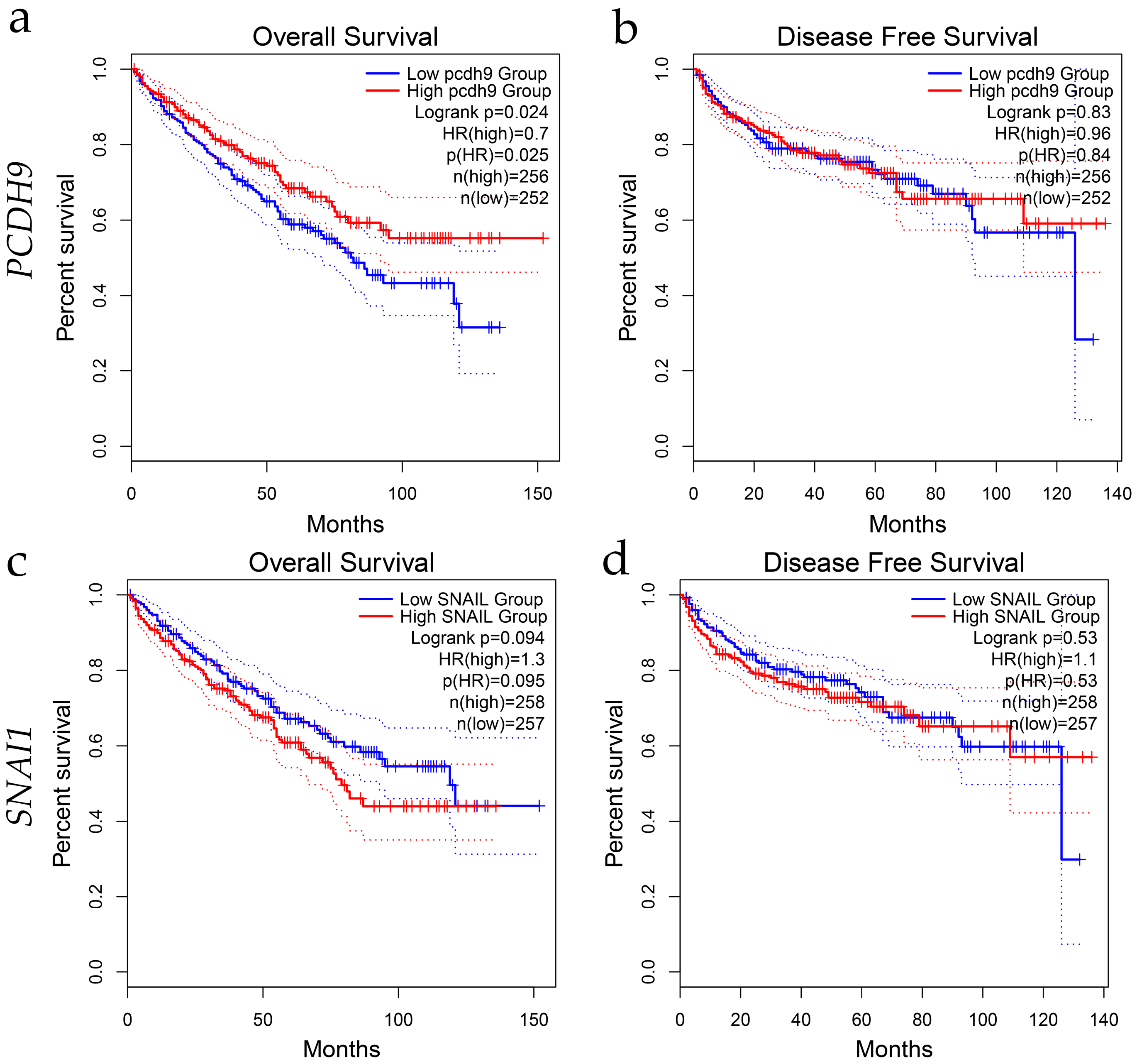

Figure S1. Kaplan-Meier survival analysis of PCDH9 and EMT-related genes in ccRCC. Overall survival (OS; left panels) and disease-free survival (DFS; right panels) curves for ccRCC patients stratified by median expression of PCDH9 (a,b) and SNAI1 (c,d). High expression groups are shown in red; low expression groups are shown in blue. Dotted lines represent 95% confidence intervals. (a) High PCDH9 expression is associated with significantly improved OS (log-rank p = 0.024; HR=0.7). (b) PCDH9 expression shows no significant association with DFS (log-rank p=0.83; HR=0.96). (c,d) SNAI1 expression is not significantly associated with OS (p = 0.094; HR=1.3) or DFS (p=0.53; HR=1.1). Data were obtained from GEPIA2 using the TCGA-KIRC dataset (http://gepia2.cancer-pku.cn/).

https://doi.org/10.3390/jpm16060279

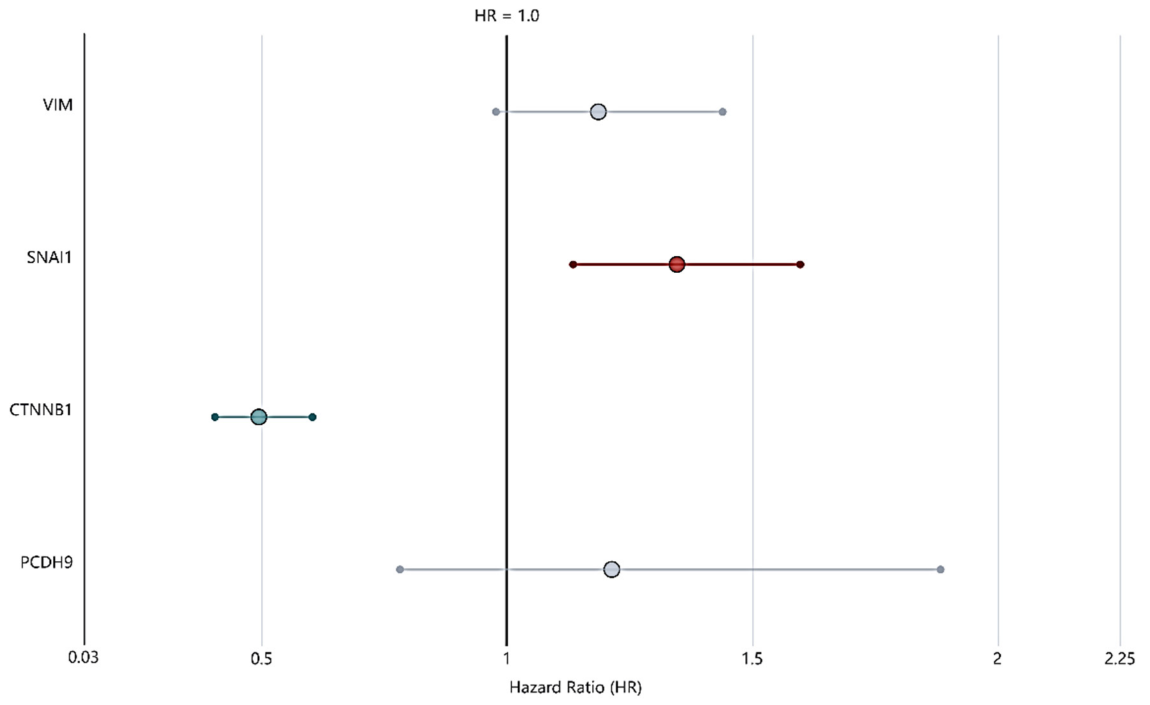

Figure S2. Multivariate Cox regression analysis of PCDH9 and EMT-related genes in ccRCC — overall survival (OS) model. Forest plot displaying hazard ratios (HR) and 95% confidence intervals (CI) from multivariate Cox proportional hazards regression analysis using OS as the endpoint. All four genes (VIM, SNAI1, CTNNB1, PCDH9) were included simultaneously in the model. CTNNB1 remains strongly protective (HR = 0.494, 95% CI: 0.405–0.603, p = 4.71 × 10−12). SNAI1 is significantly associated with increased mortality (HR = 1.347, 95% CI: 1.135–1.598, p = 0.00065). VIM shows a non-significant trend (HR = 1.186, p = 0.0835). PCDH9 is not significant (HR = 1.214, p = 0.387). The vertical line indicates HR = 1.0 (no effect). Points with confidence intervals entirely to the left of HR = 1.0 indicate a protective effect (teal); points entirely to the right indicate increased risk (red); overlapping HR = 1.0 indicates non-significance (grey). Data were obtained from GEPIA3 using the TCGA-KIRC dataset (http://gepia3.cancer-pku.cn/).

https://doi.org/10.3390/jpm16060279
